# Experimental Adaptation of Murine Norovirus to Calcium Hydroxide

**DOI:** 10.3389/fmicb.2022.848439

**Published:** 2022-03-31

**Authors:** Wakana Oishi, Mikiko Sato, Kengo Kubota, Ryoka Ishiyama, Reiko Takai-Todaka, Kei Haga, Kazuhiko Katayama, Daisuke Sano

**Affiliations:** ^1^Department of Civil and Environmental Engineering, Graduate School of Engineering, Tohoku University, Sendai, Japan; ^2^Department of Frontier Sciences for Advanced Environment, Graduate School of Environmental Studies, Tohoku University, Sendai, Japan; ^3^Laboratory of Viral Infection I, Department of Infection Control and Immunology, Ōmura Satoshi Memorial Institute & Graduate School of Infection Control Sciences, Kitasato University, Tokyo, Japan; ^4^Research Institute for Humanity and Nature, Kyoto, Japan

**Keywords:** murine norovirus, experimental evolution, disinfection, slaked lime, calcium hydroxide

## Abstract

Slaked lime (calcium hydroxide) is a commonly used disinfectant for fecal sludge. Although viruses are inactivated by lime treatment, whether RNA viruses adapt to lime treatment has not yet been determined. Here, we show that murine norovirus developed higher tolerance during serial passages with lime treatment. We compared synonymous and non-synonymous nucleotide diversities of the three open reading frames of viral genome and revealed that virus populations were subjected to enhanced purifying selection over the course of serial passages with lime treatment. Virus adaptation to lime treatment was coincident with amino acid substitution of lysine to arginine at position 345 (K345R) on the major capsid protein VP1, which accounted for more than 90% of the population. The infectious clones with the K345R produced using a plasmid-based reverse genetics system exhibited greater tolerance in a lime solution, which indicated that the specific amino acid substitution was solely involved in the viral tolerance in lime treatment.

## Introduction

Norovirus, a member of family *Caliciviridae*, is known as a major cause of acute gastroenteritis. According to a systematic review of 175 studies, the pooled prevalence of norovirus in 187,336 patients with acute gastroenteritis was 18% (95% CI:17–20%) worldwide (Ahmed et al., 2014). Norovirus is shed primarily in the stool but also can be found in the vomitus of infected persons, and transmission occurs *via* fecal–oral and vomit–oral pathways by four general routes: direct person-to-person, foodborne, waterborne, and through environmental fomites (Lopman et al., 2012; de Graaf et al., 2016). Noroviruses are detected from excreta, untreated wastewater (Katayama and Vinjé, 2017), and treated wastewater used for irrigation (Adegoke et al., 2018). Previous studies have reported gastroenteritis outbreaks due to the consumption of crops contaminated by noroviruses (Falkenhorst et al., 2005; Ethelberg et al., 2010).

In a non-sewered sanitation system, human excreta is stored in a pit or container at least 1 year to make the material safe to handle (WHO, 2015; Oishi et al., 2021). Without this long-term storage, farm workers and sanitation workers can be directly exposed to viruses by oral transmission during collection, irrigation, and application of the excreta to farmland (Julian et al., 2018; Bischel et al., 2019). The infection risk in the use of organic fertilizer is reduced by adequate sanitization of human excreta. Lime is commonly used as a base disinfectant for high organic loading waste, including feces, fecal sludge, and compost. Slaked lime [Ca(OH)_2_] and quick lime (CaO) are dominant types of the lime disinfectant, because they are highly reactive and generate hydroxide ions, which are strong bases (Kohn et al., 2017). Lime treatment raises the matrix pH above 10, at which most microorganisms, including surrogate phages (Hijikata et al., 2016; Ogunyoku et al., 2016) and murine norovirus (MNV) (Sangsriratanakul et al., 2018), cannot survive.

Requirements for disinfection conditions (e.g., disinfectant dosage and treatment time) are determined on the basis of the tolerances of reference viruses in a disinfection study. Meanwhile, previous studies have reported the heterogeneous susceptibilities of reference viruses and indicators for waterborne enteric viruses within and among genetically relative strains. For example, disinfection studies of the *Enterovirus* genus have demonstrated that the tolerance to free chlorine, ClO_2_, UV_254_, and ozone varied among serotypes and among strains in the serotype coxsackievirus B5 (Meister et al., 2018; Torii et al., 2021). These studies also established that environmental strains are less sensitive to disinfection compared to laboratory strains (Meister et al., 2018; Torii et al., 2021). Similarly, the tolerance to free chlorine varied among environmental strains of F-RNA phage genotype I (GI) and laboratory strains of GI phage (Torii and Katayama, 2020).

The varied sensitivities in RNA viruses and some DNA viruses are explained by their distinctive features, high mutation rates, and continuous production of variant genomes that provide a rich substrate for selection and random drift to act on (Domingo et al., 2012). A viral quasispecies is a complex and dynamic alternation of mutant swarms in a population that has explained the adaptive potential of RNA viruses to disinfection. The highly adaptive potential of RNA viruses can lead to the emergence of the less sensitive populations that are not sufficiently inactivated in the disinfection condition determined by the sensitivity of a laboratory strain. To develop an effective measure to inactivate the less sensitive viruses, we need to characterize the phenotypic/genetic traits of less sensitive viruses and clarify the adaptation mechanisms against disinfection stress.

Murine norovirus, which belongs to the genus *Norovirus*, is used as a model virus for human norovirus in disinfection studies on inactivation efficiency and mechanisms (Park et al., 2016; Maffettone et al., 2020) and adaptation to disinfectants (Rachmadi et al., 2018). Each of the four open reading frames (ORFs) in the viral genomic RNA encodes non-structural (NS) proteins, the major capsid protein (VP1), the minor structural protein (VP2), and a protein called virulence factor 1 (VF1) (Mauroy et al., 2017). A previous study identified a unique non-synonymous mutation in ORF3 of the chlorine-treated populations but showed that free chlorine disinfection exerted a selection pressure for MNV (Rachmadi et al., 2018). Sangsriratanakul et al. (2018) studied the viral inactivation efficiency in lime solutions, but the adaptation to lime treatment and the evolutional mechanisms have not yet been studied.

This study aims to characterize the phenotypic/genetic traits of norovirus through adaptation to lime treatment. To this end, we adapted a viral population from MNV S7 to lime treatment by serial passage with exposure to a slaked lime solution [6.8 mM Ca(OH)_2_, pH 12.0–12.2, at 22°C]. The underlying evolutionary mechanisms for virus adaptation were presumed on the basis of the metrics for virus genetic diversity. We conducted the lime sensitivity assay using the infectious clones produced *via* a plasmid-based reverse genetics system to assess whether a specific mutation led to a higher tolerance to lime treatment.

## Materials and Methods

### Murine Norovirus and Cell Lines

Prof. Yukinobu Tohya, Nihon University, Japan, provided the MNV S7 PP3 strain. MNV S7 PP3 was propagated, enumerated, and purified according to the published protocols. RAW 264.7 cells [American Type Culture Collection (ATCC) TIB-71] were used as host cells and were cultured in Dulbecco’s Modified Eagle Medium (Nissui) containing 10% (v/v) fetal bovine serum (Gibco), 0.1% (w/v) NaHCO_3_, 2 mM L-glutamine, 10 mM non-essential amino acids, penicillin (100 mg/ml), and streptomycin (100 U/ml).

### Serial Passages of Murine Norovirus With and Without Lime Treatment

**FIGURE 1:**
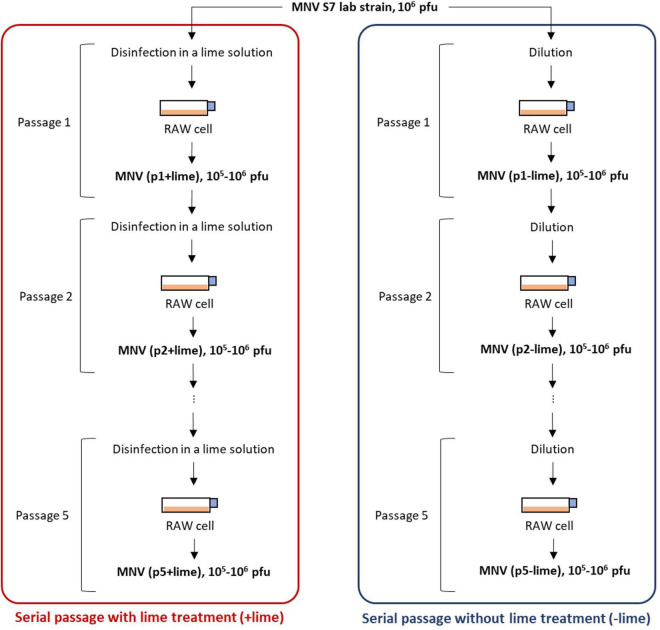
Schematic depiction of serial passages as performed in experimental adaptation of murine norovirus (MNV). Once an initial infection is completed in plate 1, the virus is titrated and the desired amount is used to infect the cells in plate 2. The process was repeated five times.

A virus population was taken from a laboratory strain of MNV S7 PP3 as an ancestor of serial passages with and without lime treatment (Figure 1). MNV populations derived from the *n*th passage without lime treatment (−lime) and with lime treatment (+lime) were designated as p*n* + lime and p*n*−lime virus, respectively (*n* = 1, 2, 3, 4, and 5).

The lime solution was prepared by dissolving 15 mg of calcium hydroxide (Fujifilm-Wako, Osaka, Japan) in 30 ml of sterilized pure water so that the pH value increased to approximately 12. A 100-μl p*n* + lime MNV suspension in the culture medium [10^6^–10^7^ plaque forming unit (PFU)/ml] was exposed to 900 μl of lime solution at room temperature (22°C) and inactivated to 10^3^ PFU/ml. The treatment time for less sensitive populations was prolonged so that the infectious titer would be 10^3^ PFU/ml after treatment. A 100-μl p*n*−lime MNV suspension in the culture medium was diluted with the culture medium to create 10^3^ PFU/ml, in parallel. Each of the virus suspension was inoculated onto the confluent RAW cells cultured in T75 flask containing 25 ml of the culture medium [the multiplicity of infection (MOI) was approximately 10^–4^]. The MNV was incubated at 37^°^C with 5% CO_2_ for 3 days. The cell suspension was subjected to three rounds of freeze-thawing and then transferred to a 50-ml falcon tube. The falcon tube was centrifuged at 10,000 *g* for 10 min at 4^°^C, and the supernatant was passed through a PolyVinylidene DiFluoride (PVDF) membrane filter (0.2-μm pore). The collected MNV suspension, p*n*+1+lime and p*n*+1−lime, was exposed to the lime solution or diluted with the culture medium and then inoculated onto the confluent RAW cells (Figure 1). These processes were repeated five times for each passage type. The entire selection process was repeated twice. The first and second selections were referred to as the first and second rounds, respectively. The obtained MNV suspensions were stored at −80^°^C until the disinfection experiment and next generation sequencing (NGS) library preparation.

### Evaluation of Lime Sensitivity

The lime sensitivity of viruses was evaluated on the basis of the reduction of infectious titers by exposure to lime solutions. The lime sensitivity assay was conducted in triplicate for each sample. MNV suspension (100 μl) in the cultured medium was added to a 1.5-ml tube containing 900 μl of the lime solution. Experiments were carried out at 22°C. In the sensitivity assay for the populations in −lime and +lime lineages, samples were periodically taken during the 5-min exposure to the lime solution and immediately diluted with the culture medium. In the sensitivity assay for the plaque-purified isolates and the infectious clones, samples were taken at 4 min because MNV was not always detected after the longer exposure time.

Murine norovirus infectivity was determined by plaque assays in the sensitivity assay for the populations in −lime and +lime lineages. One milliliter of the sample was inoculated onto the confluent RAW cell cultured in the six-well plate and incubated at 37°C with 5% CO_2_ for 90 min with periodical swaying. The virus suspension was removed after the 90-min incubation, and 2 ml of the agar medium (1.25% agar) were added. The six-well plates were incubated for 2 days at 37°C with 5% CO_2_ and then dyed with 0.03% neutral red solution and incubated for 2–3 h. The neutral red solution was removed and the six-well plates were incubated for 1 h, and then, the plaques were counted.

Murine norovirus infectivity was determined by Median Tissue Culture Infectious Dose (TCID_50_) assays in the sensitivity assay for the plaque-purified isolates and the infectious clones produced using the plasmid-based reverse genetics system. We performed the TCID_50_ assays because these clones and isolates did not form clear plaques with unknown reasons. We did not compare the data obtained using the two different methods for titration, and we evaluated the lime sensitivity within the data obtained from identical method. Three-fold serial dilutions of virus were prepared in 96-well plates containing culture medium. Fifty microliters of the RAW cell suspension were inoculated in each well. The 96-well plates were incubated at 37°C with 5% CO_2_. After 2 days, a cytopathic effect was observed with light microscopy. TCID_50_ values were calculated according to the Spearman and Kärber algorithm (Hierholzer and Killington, 1996).

### Plaque Purification

The populations obtained from the +lime and −lime lineages were inoculated onto the confluent RAW cells cultured in six-well plates (MOI of 10^–4^). Plaques were formed using the method described above. Well-separated plaque from any other plaques was taken using 1,000 μl of barrier tips cut by a sterilized cutter, placed into a 1.5-ml tube containing 500 μl of culture medium, and vortexed. The isolate suspensions were incubated overnight at 4°C and then passed through a membrane filter (0.2-μm pore). The first plaque-purified virus suspension was subjected to a second round of plaque purification. After the third round of plaque purification, virus suspensions were stored at −80°C until the subsequent analysis. Finally, we obtained six isolates originated from the p5 + lime populations, six isolates originated from the p5−lime populations, and three isolates originated from the ancestor population in the experimental adaptation (Supplementary Figure 1). Each of 15 MNV isolates was amplified in a single passage at MOI of 10^–4^ on the confluent RAW cells to increase the viral titer for sensitivity assays and NGS library preparation.

### Construction of Plasmid for Production of Infectious Clones

The construct pMNV_*A*6089*G*_ plasmid was generated from pMNV S7 (Katayama et al., 2014) by introducing mutations detected in the ancestor virus and the less sensitive viruses. The In-Fusion system (Takara) was used for fragment insertion into plasmid DNA. PrimeSTAR max DNA Polymerase (Takara) was used for mutagenesis of pMNV S7. Primers used for the In-Fusion reaction and mutagenesis are listed in a table in Supplementary Figure 2.

### Transfection of pMNV_*A*6089*G*_ to HEK293T

Infectious clones (referred to as clone MNV) were produced using a plasmid-based reverse genetics system as described previously (Katayama et al., 2014). HEK293T cells cultured in a six-well plate with 2 ml of culture media were transfected with pMNV_*A*6089*G*_ using Lipofectamine 2000 (Invitrogen). Supernatants from transfected cultures were collected at 48 h after transfection and inoculated onto Huh7.5.1 CD300lf cells cultured in six-well plates. The virus was passaged three times in Huh7.5.1 CD300lf cells to obtain a sufficient titer for disinfection experiments. The whole genome sequence of propagated virus was confirmed using next-generation sequencing.

### Whole Genome Sequencing

Viral RNA was extracted from the MNV suspension using the QIAamp Viral RNA Mini kit (Qiagen, Germany) according to the manufacturer’s instructions. Ribosomal RNA (rRNA) in RNA suspension was removed using the NEBNext rRNA Depletion Kit (Human/Mouse/Rat) (New England, Ipswich, MA, United States). A 300-bp fragment library was constructed for each sample using the NEBNext Ultra II RNA Library Prep Kit for Illumina according to the manufacturer’s instructions. Samples were added bar codes for multiplexing using the NEBNext Multiplex Oligos for Illumina (Dual Index Primers Set 1). Library purification was done using Agencourt AMPure XP magnetic beads (Beckman Coulter, Pasadena, CA, United States) according to the NEBNext Protocol. The quality of the purified libraries was assessed on an Agilent 2100 Bioanalyzer using a High Sensitivity DNA Kit (Agilent Technologies, Santa Clara, CA, United States). The concentration of the purified library was determined on a Qubit 2.0 fluorometer using the Qubit HS DNA Assay (Invitrogen, Carlsbad, CA, United States) and the NEBNext Library Quant Kit for Illumina. Libraries generated from the populations obtained in serial passages, plaque purified isolates, and infectious MNV were sequenced with paired-end 150-bp reads on an Illumina NovaSeq 6000, paired-end 250-bp reads on an Illumina MiSeq, and paired-end 150-bp reads on an Illumina iSeq (Illumina, San Diego, CA, United States), respectively.

### Bioinformatic Analysis

The FASTQ formatted sequence data were analyzed using CLC Genomics Workbench Software version 10.1.1 (CLC Bio, Aarhus, Denmark). The *Trim Sequences* function trimmed index primer sequences used for library amplification and sequencing. The MNV S7 PP3 genome sequence obtained from National Center for Biotechnology Information (NCBI) (GenBank: AB435515.1) was used as the reference sequence. The trimmed sequence reads were aligned to the reference sequence using the *Map Reads to Reference* function, and then realigned using the *Local Realignment* function. Sequence coverages were obtained by the locally realigned read files to SAMtools (Danecek et al., 2021). The consensus sequence of the MNV laboratory strain (the ancestor population in the serial passages) was determined using the *Extract Consensus Sequence* function, so that it was used as a reference sequence for the variant detection for the populations amplified through the serial passages.

For detection of the single-nucleotide polymorphisms (SNPs) in the MNV isolates, the MNV S7 PP3 genome sequence (GenBank: AB435515.1) was used as a reference sequence. The frequency of SNPs was determined using the *Low Frequency Variant Detection* function. The threshold value of the error rate was set to 1% to avoid the false positive of variant detection. SNPs with a coverage depth of at least 10 were considered according to the default setting of the CLC Genomics Workbench. Genome-wide and site-specific nucleotide diversities (π_*S*_ and π_*N*_) were calculated with SNPGenie (Nelson et al., 2015).

### Regularized Regression Modeling

We performed a regularized regression modeling to evaluate the significance of SNPs for the viral lime sensitivity using the NGS data and sensitivity data of the MNV isolates. We considered the following multiple linear regression model:


(1) y=Xβ+ε

where ***y*** is the logarithm of the inactivation ratio [the log reduction values (LRVs)] of viruses at 4 min after exposure to lime solutions, **β** is a regression coefficient, and **ε** is the residuals. The vectors ***y*** and **ε** consist of *n* elements that correspond to the number of samples with a set of the LRVs and SNPs. The vector **β** consists of *p* + 1 elements that are allocated to *p* regression coefficients and one intercept. X is a *n* × (p + 1) design matrix that includes the frequency of SNPs at *p* polymorphic sites in *n* samples. We assumed that the value of β_*i*_ indicated the contribution of the SNPs at site *i* in the viral tolerance to lime treatment. The Elastic Net (EN) method (Zou and Hastie, 2005) was employed to estimate **β** in Eq. 1 as follows:


(2) $$\hat{\beta }=argmin_{\beta }\left({||\text{y}-\text{X}\,\beta ||}_{2}^{2}+\lambda \,\left(\alpha \,{||\beta ||}_{1}+\left(1-\alpha \right)\,{||\beta ||}_{2}^{2}\right)\right)$$

where λ(λ > 0) is a regularizing parameter and α(0 < α < 1) is a penalty weight for regularization. The EN regression was performed by the glmnet package (Hastie and Qian, 2016) on the R software (version 3.6.1) (R Development Core Team, 2019). The value of λ was determined by *k*-fold cross-validation to minimize the mean square value plus standard error (minMSE+1SE).

## Results

### Adaptation of Murine Norovirus to Lime Treatment

**FIGURE 2:**
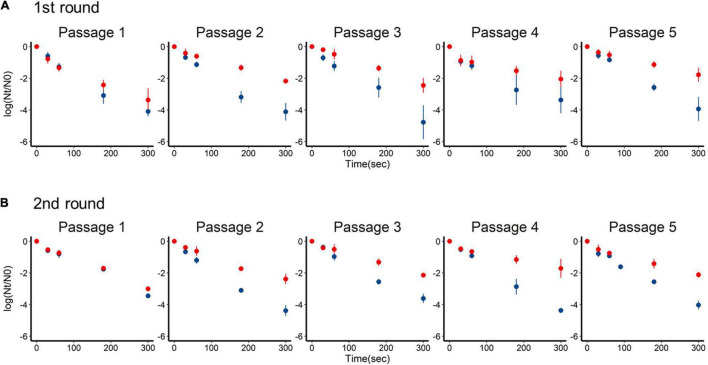
Time-series inactivation rate of murine norovirus obtained in serial passages without (blue) and with (red) lime treatment in the first **(A)** and second **(B)** rounds. Each point represents an average value from three replicates.

**FIGURE 3:**
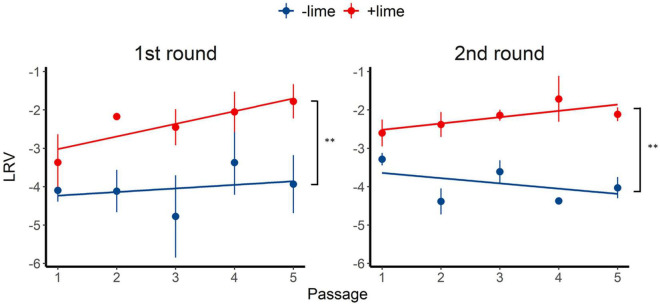
Inactivation rate of murine norovirus (log reduction value: LRV) obtained in serial passages without (−lime) and with (+lime) lime treatment 5 min after exposure to lime solution. Each point represents an average value from three replicates. Solid lines are linear regression lines of best fit. Asterisks indicate a statistically difference between the −lime lineages and +lime lineages (*p* < 0.01 by Mann–Whitney *U* test).

Inactivation of the +lime and −lime populations is shown in Figure 2 as a function of treatment time. The LRVs at 5 min of the p1 + lime and p1−lime were 3.0–4.2 and 2.5–3.5, respectively (Figure 3). The LRVs of the −lime populations did not change across the five passages (*p* = 0.55 and *p* = 0.15 in the first round and the second round, by linear regression non-zero slope *T*-test; Figure 3). Meanwhile, the LRVs of the +lime populations got smaller with each passage (*p* = 0.0048 and *p* = 0.031 in the first and second round, by linear regression non-zero slope *T*-test; Figure 3). The mean LRVs were significantly different between the +lime and −lime populations (*p* = 2.5 × 10^–5^ and *p* = 5.1 × 10^–6^ in the first and second rounds, respectively, using the Mann–Whitney *U* test).

### Nucleotide Diversity

**FIGURE 4:**
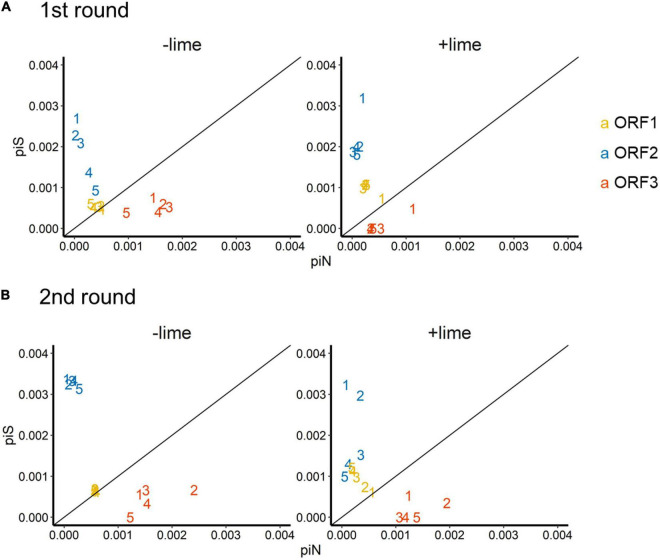
Time series of nucleotide diversity at non-synonymous sites (piN) and synonymous sites (piS) in each protein coding gene [open reading frames (ORFs) 1, 2, and 3] in the first **(A)** and second **(B)** rounds. Each passage time is described as the number on panels. Solid lines represent the 1:1 relationship between piS and piN indicative of natural selection and neutral evolution.

The depth of coverage was greater than 300 reads across the entire coding region for 99.9% of the sequencing libraries. We estimated synonymous nucleotide diversity (π_*S*_) and non-synonymous nucleotide diversity (π_*N*_) for the three protein coding genes (ORFs 1, 2, and 3) separately (Figure 4).

**FIGURE 5:**
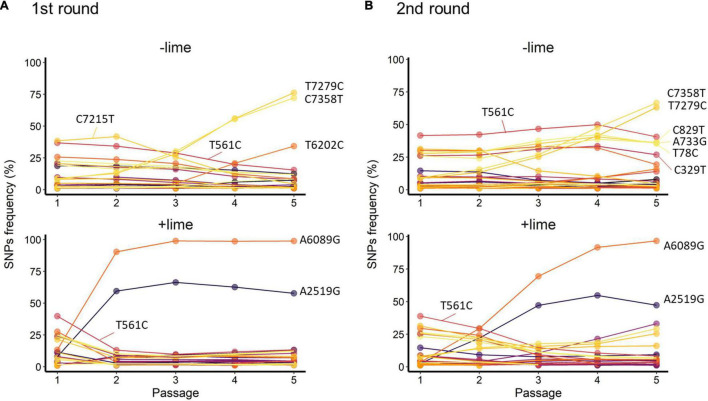
Trajectories of single-nucleotide polymorphisms (SNPs) detected with at least 1% frequency through the serial passage with (+lime) and without (−lime) treatment in the first **(A)** and second **(B)** rounds.

For ORF1 in the −lime populations, the π_*S*_ and π_*N*_ were within the 10^–4^ order, and the π_*N*_ /π_*S*_ values were close to 1, whereas, in the +lime populations, π_*S*_ increased and π_*N*_ decreased over the course of serial passages (Figure 4). For ORF2 in the +lime and −lime populations, the π_*N*_ /π_*S*_ values were less than 1, whereas π_*N*_ got larger with each passage in the −lime populations (Figure 4). In the +lime populations, in contrast, π_*N*_ got close to zero after passage 2, whereas the unique non-synonymous nucleotide change (A6089G) accounted for over the 90% of the virus populations (Figure 5). ORF3 in the +lime populations had no synonymous mutations, and π_*S*_ values were zero after passages 2 and 3, in the first and second rounds, respectively. Meanwhile, non-synonymous mutations were detected in all populations, and accordingly the π_*N*_ /π_*S*_ values were greater than 1 (Figure 4).

### Major Single-Nucleotide Polymorphisms in the Murine Norovirus Populations

Single-nucleotide polymorphisms called at greater than 1% frequency, and at least 300 reads of coverage were used for the analysis. Four non-synonymous mutations were detected in the laboratory strain with > 99% frequency: G1564A (ORF1), C6289G (ORF2), T6605C (ORF2), and T6751C (ORF3). The consensus sequence of the ancestor population was used as the reference sequence for the SNPs detection from populations obtained in the serial passages.

T561C and T7279C accounted for over 35% of the −lime populations (Figure 5). T561C and T7279C were recurrences of the MNV S7 PP3; the origin of our laboratory strain and, thus, the two SNPs could be common variants in the viral population of the MNV S7 PP3. They were fixed as a consequence of several bottleneck events. The trajectories of the C7215T in the first round and four SNPs (C829T, A733G, A78C, and C329T) in the second round were parallel with T561C, whereas those of the two SNPs (C7358T and T6202C) in the first round and TC7385T in the second round were parallel with T7279C (Figure 5). The parallel trajectories with the common variants indicated that those SNPs were the consequence of genetic hitchhiking with T561C and T7279C.

In the +lime lineages, one synonymous SNP (A2519G) and one non-synonymous SNP (A6089G) arose in the populations with high frequency (Figure 5). In the first round, the frequency of A6089G increased from less than 0.5% to greater than 90% by passage 2, and increased to 98.8% by passage 5 in the first round. In the second round, the frequency of A6089G was less than 35% by passage 2 and increased to 96.6% by passage 5. The mutation A6089G associated with the amino acid substitution of lysine (K) to arginine (R) at site 345 on VP1. We confirmed the location of K345R by assigning the nucleotide changes to the sequence of MNV S7 PP3 reported by Nelson et al. (2018) and visualized using PyMOL (Schrödinger LLC, 2015). The K345R is located on the P2 subdomain, specifically on the opposite side of the contact site to the receptor CD300lf (Supplementary Figure 3).

A2519G and A6089G did not demonstrate parallel trajectories indicative of genetic hitchhiking. In the first round, the frequency of A2519G increased to 66.4% by passage 3, and, similarly, in the second round, the frequency of A2519G increased to 54.7% by passage 4 (Figure 5). The trajectories of A2519G were reproductive in the populations from the +lime lineages, which indicated that A2519G frequency was increased due to the lime treatment.

### Single-Nucleotide Polymorphisms in Association With Lime Sensitivity

The frequency of A6089G and A2519G was associated with the greater tolerance of virus populations to the lime treatment, and thus, we hypothesized that those two SNPs contributed to the lime sensitivity of MNV. To verify this hypothesis, we increased the sample size by plaque isolation from the sensitive and less sensitive MNV populations and evaluated the association between the SNPs frequency and the LRVs of the isolates.

The LRVs were smaller in the isolate originated from the less sensitive populations (J, K, L, M, N, and O) compared to the other isolates (Supplementary Figure 4). The less sensitive six isolates shared A6089G, and the LRVs were significantly smaller in the isolates with the A6089G mutation (*p* = 0.027 by Mann–Whitney *U* test; Supplementary Figure 4). More SNPs were identified in the 15 isolates compared to the founder virus populations (Supplementary Figure 5). Although the 15 isolates shared the five major SNPs (G1564A, C6289G, T6605C, T6751C, and C7112T), the appearances of polymorphic sites were not consistent across the isolates (Supplementary Figure 5). Less sensitive populations were purified in the plaque-to-plaque transfer, whereas the genetical heterogeneity increased during the replication in the cultivation conducted before the NGS library preparation because mutations could be induced in each replicate. The A6089G remained in the isolates from the less sensitive populations, but A2519G was not detected among all the less sensitive isolates (Supplementary Figure 5). In this study, the coefficient values in the EN regression indicate the significance of SNPs for the viral tolerance to lime treatment. The coefficient value that corresponded to A6089G was not zero, but that for other SNPs was estimated to be zero (Supplementary Figure 6), which indicated that A6089G was important for the viral tolerance in an alkali environment, whereas A2519G was not.

**FIGURE 6:**
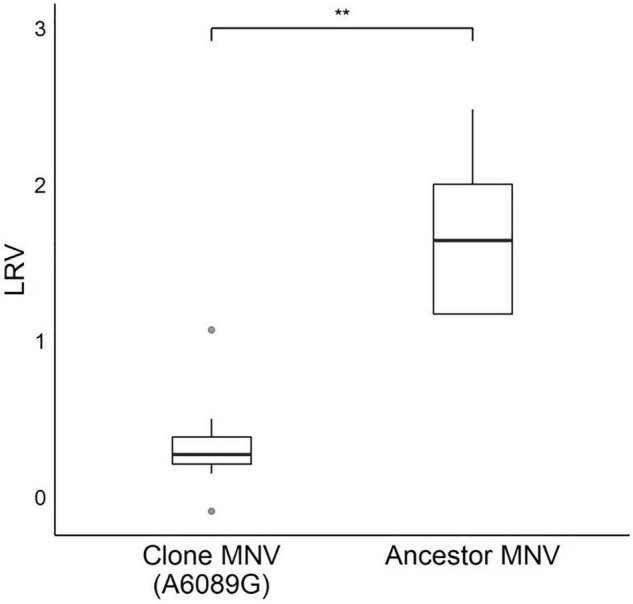
Log reduction values (LRVs) of infectious particles produced using a plasmid-based reverse genetics system [clone MNV (A6089G)] and the laboratory strain used as the founder in the serial passages with lime treatment (ancestor MNV). Asterisks indicate a statistically difference between the two viruses (*p* < 0.01 by Mann–Whitney *U* test).

The sole effect of A6089G on the lime sensitivity was verified by comparing the LRVs of infectious clones produced using a plasmid-based reverse genetics system (clone MNV) and ancestor virus used for the serial passage with lime treatment. Those two populations shared non-synonymous mutations on ORF2 (C6289G and T6605C) and ORF3 (T6751C), whereas clone MNV had a specific mutation on ORF2 (A6089G). The LRVs were significantly smaller in clone MNV than ancestor virus (*p* = 0.004 by Mann–Whitney *U* test; Figure 6), which indicated that A6089G contributed solely to the viral tolerance to lime treatment.

## Discussion

We performed an experimental adaptation of MNV and demonstrated that the MNV lineages adapted to the lime treatment acquired greater tolerance, whereas the lineages cultured without the lime treatment did not. All the +lime and −lime populations evolved under the bottleneck due to the different processes (dilution and disinfection). Data obtained in the disinfection experiment suggested that viral populations evolved by different mechanisms in addition to bottleneck constraints, which led to different sensitivities to the lime treatment. The LRVs of the +lime populations decreased with the increase in frequency of A6089G and A2519G (Figures 2, 5). A sensitivity assay using the infectious clones produced using the plasmid-based reverse genetics system allowed us to identify the specific mutation uniquely responsible for the increased tolerance to the lime treatment (Figure 6).

To clarify the underlying virus evolutional mechanisms in the lime treatment, the relative indices of π_*S*_ and π_*N*_ were compared between the +lime and −lime populations (Figure 4). The π_*N*_/π_*S*_ value is used to assess natural selection, with values greater than 1 indicative of positive selection and less than 1 indicative of purifying selection (Nelson and Hughes, 2015). For ORF3, we found no clear trends in non-synonymous diversity and synonymous diversity between the +lime and −lime populations (Figure 4), which indicated that VP2 was not important for adaptation to the lime treatment. Previous studies suggest that VP2 is involved in virion stability (Glass et al., 2000; Bertolotti-ciarlet et al., 2003) and receptor engagement (Conley et al., 2019). The reason the nucleotide diversity of ORF3 is not affected by the lime treatment remains unclear, but it may suggest lower structural constraints of VP2 in a lime solution. The number of mutations was smaller in ORF3 compared to ORF1 and ORF2 (data not shown), which was contrasted to the previous report on MNV-1 that demonstrated a larger number of mutations in a region in the ORF3 (35 non-synonymous and 22 synonymous) compared to the other genes (Mauroy et al., 2017). The number of mutations in the ORF3 may increase if we proceed more than five times passages. Meanwhile, our result was similar to a previous study on MNV S7 PP3, which observed more variations in the anterior coding region of ORF1 and very few in the ORF3 and 3’UTR regions throughout adaptation to RAW cells (Kitamoto et al., 2017). These results indicate that the degree of conservation varies among MNV strains, and thus, MNV-1 may exhibit different evolutionary mechanisms during serial passages with the lime treatment.

Populations from the +lime and −lime lineages were distinguished for the ORF1 and ORF2 diversity. For ORF1, the π_*N*_/π_*S*_ values of the −lime populations were close to 1, whereas, in the +lime populations, π_*S*_ increased while π_*N*_ decreased over the course of serial passages (Figure 4), which indicated that the evolution of NS proteins was neutral in the −lime lineages. In contrast, the purifying constraint was reinforced in the latter passages in the +lime lineages. The NS proteins are involved in viral replication and integration of several precursor proteins (Thorne and Goodfellow, 2014). The decline in π_*N*_ of ORF1 could result in fewer variations in the NS protein in the +lime populations. If amino acid substitutions are not allowed in the NS proteins of the viral population, then the replication fidelity may become consistent in a population, which leads to effective production of viruses with uniform characteristics, including adaptiveness to an alkali environment.

For ORF2, π_*N*_ was smaller than π_*S*_ in the +lime and −lime populations, which indicated that ORF2 was highly important to viral fitness, and VP1 was under the purifying constraints during serial passages regardless of the lime treatment (Nelson and Hughes, 2015). In the −lime populations, π_*N*_ became larger with each passage, which indicated that the purifying constraint was relaxed through the repeated bottleneck event without the lime treatment. In the +lime populations, in contrast, π_*N*_ got close to zero after passage 2. These results suggested that the highly adaptive MNV populations evolved under the purifying constraints on VP1 and NS proteins provided by the lime treatment. Over 90% of the adaptive populations fixed A6089G, but other non-synonymous mutations were not fixed in ORF2. The sole contribution of A6089G to the viral tolerance to the lime treatment was verified according to the sensitivity of clone MNV produced using a plasmid-based reverse genetics system. The norovirus capsid consists of 180 VP1 molecules paired with 90 dimers that self-assemble to form a *T* = 3 icosahedral structure with a diameter of 35–39 nm, which was confirmed with recombinant Norwalk virus particles (Prasad et al., 1994). The K345R is located on the opposite side of the contact site to the receptor CD3001f on the P2 subdomain (Nelson et al., 2018; Supplementary Figure 6), which comprises the digital surface of the virion and is a target for neutral antibodies (Tohya et al., 1997; Nelson et al., 2018). The K345R could affect the surface charge of the capsid, which was involved in the stability of MNV in the lime solution, due to the different electrostatic property between lysine and arginine. Notably, the pK_3_ values are 10.67 in lysine and 12.10 in arginine at 25°C (Atkins and de Paula, 2011). Although the pK_3_ is affected by the temperature and ionic strength of a solution, lysine could have a minus charge and arginine could have relatively neutral charge in the alkali pH set in this study. The difference in the charge of a single amino acid on a monomer can result in a significant difference in the electrostatic property of the composed MNV capsid, which can affect interlinkages among amino acids within a monomer as well as the interaction between individual viruses.

A possible cause of the lower sensitivity is that the substitution of lysine to arginine increases the stability of the MNV capsid in alkali solutions, and the less sensitive population consists of less sensitive individuals that are robust to the electrostatic repulsion force within a capsid protein (Supplementary Figure 7A). Another hypothesis is that the change of surface charge due to amino acid substitution enhances the formation MNV aggregates in lime solutions, which results in more MNVs included inside the aggregate and can remain intact (Supplementary Figure 7B). The capsid structure of MNV changes in response to the pH of an aqueous solution, which affects infection efficiency (Song et al., 2020), and thus, it could happen in the lime solution used in this study. The human norovirus-like particles are denatured in alkali solutions, which results in the formation of viral aggregates (Sato et al., 2016). Formation of viral aggregates reduces the disinfection efficiency by consumption of disinfectant within the aggregate (Gerba and Betancourt, 2017). Viruses inside a viral aggregate can escape from disinfection, due to the consumption of effective virucidal agent by viruses accumulated on the outer layer of the aggregate. The intact MNV included inside the aggregate could be released in the culture medium used for dilution before plaque assay. In the above situations, more individual viruses can remain intact in an alkali environment and can replicate in the subsequent cultivation in the RAW cells or plaque assay. Calcium carbonate, which is readily formulated in the presence of carbon dioxide, may become the core of the viral aggregate in a lime solution (Supplementary Figure 7B). The above hypothesis should be validated in further examinations of the structural changes of VP1 for MNV with and without the specific amino acid substitution. Visualization tools such as cryo-electron microscopy and particle tracking analysis method can be used to clarify the MNV morphology.

Another issue of interest is whether the mutation A6089G can remain in populations after additional passages without lime treatment. Because the frequency of A 6089G was almost 99% of the less sensitive population, the mutation could remain in an aliquot of viral suspension after the reduction of population size (dilution). If A6089G is maintained due to the presence of lime, then it may disappear from populations after additional passages without lime treatment although it needs to be confirmd in future studies. Future studies also need to assess cross-resistance with other disinfectants which has been demonstrated previously (Zhong et al., 2017). Kadoya et al. (2021) suggests that multiple disinfection processes are effective to prevent viral adaptation to disinfection when disinfection works as a form of natural selection. Because the lime treatment can work as natural selection, it is appropriate to add other disinfection processes in a sanitation system to ensure the safe of human health.

This study used MNV as a surrogate of human noroviruses which is of concern in fecal sludge treatment. Although we identify the mutation in the VP1, that is responsible for the decrease in lime sensitivity of an MNV population, it remains unclear whether human noroviruses acquire the lower sensitivity by the same mechanism (i.e., a single mutation on VP1). Because genetically diverse populations of human norovirus are observed in specimens and wastewater (Kremer et al., 2011; Li et al., 2021), we can speculate that less sensitive populations of human norovirus appear in fecal sludge, and they remain in lime-treated fecal sludge. It is important to investigate the VP1 sequence of less sensitive strains found in field studies and to validate the impact of non-synonymous mutations to lime sensitivity of human noroviruses in the further study.

## Data Availability Statement

The datasets presented in this study can be found in online repositories. The names of the repository/repositories and accession number(s) can be found below: DDBJ database with accession DRA013422.

## Author Contributions

WO and DS designed the study and wrote the manuscript. WO performed the experiments, bioinformatics, and statistical analyses. RT-T and RI performed the experiments. KaK coordinated the research activity planning and execution. KH, KeK, and MS supervised the use of NGS and bioinformatic analysis. All authors critically revised the manuscript and approved the final version.

## Conflict of Interest

The authors declare that the research was conducted in the absence of any commercial or financial relationships that could be construed as a potential conflict of interest.

## Publisher’s Note

All claims expressed in this article are solely those of the authors and do not necessarily represent those of their affiliated organizations, or those of the publisher, the editors and the reviewers. Any product that may be evaluated in this article, or claim that may be made by its manufacturer, is not guaranteed or endorsed by the publisher.
