# Supplementary material for: Experimental Adaptation of Murine Norovirus to Calcium Hydroxide
Source: Front Microbiol. 2022 Mar 31;13:848439. doi: 10.3389/fmicb.2022.848439 (PMC9009222; doi:10.3389/fmicb.2022.848439)
Supplement: Supplementary file 1 [file Presentation_1.PPTX]

## Slide 1
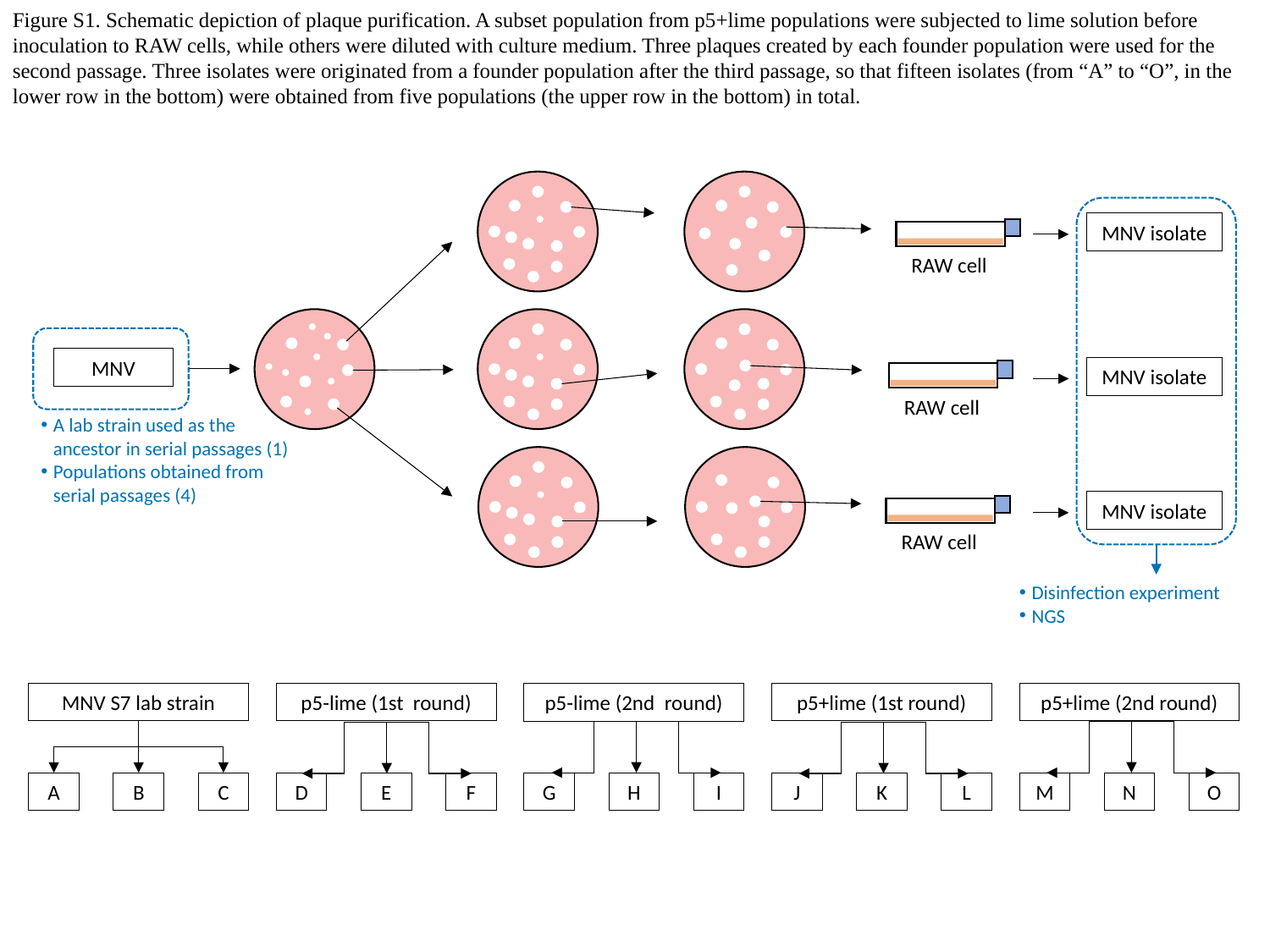

Figure S1. Schematic depiction of plaque purification. A subset population from p5+lime populations were subjected to lime solution before inoculation to RAW cells, while others were diluted with culture medium. Three plaques created by each founder population were used for the second passage. Three isolates were originated from a founder population after the third passage, so that fifteen isolates (from “A” to “O”, in the lower row in the bottom) were obtained from five populations (the upper row in the bottom) in total.
MNV isolate
RAW cell
MNV
MNV isolate
RAW cell
A lab strain used as the ancestor in serial passages (1)
Populations obtained from serial passages (4)
MNV isolate
RAW cell
Disinfection experiment
NGS
p5-lime (1st round)
p5+lime (2nd round)
MNV S7 lab strain
p5+lime (1st round)
p5-lime (2nd round)
M
N
A
B
D
O
C
E
G
H
J
K
F
I
L

## Slide 2
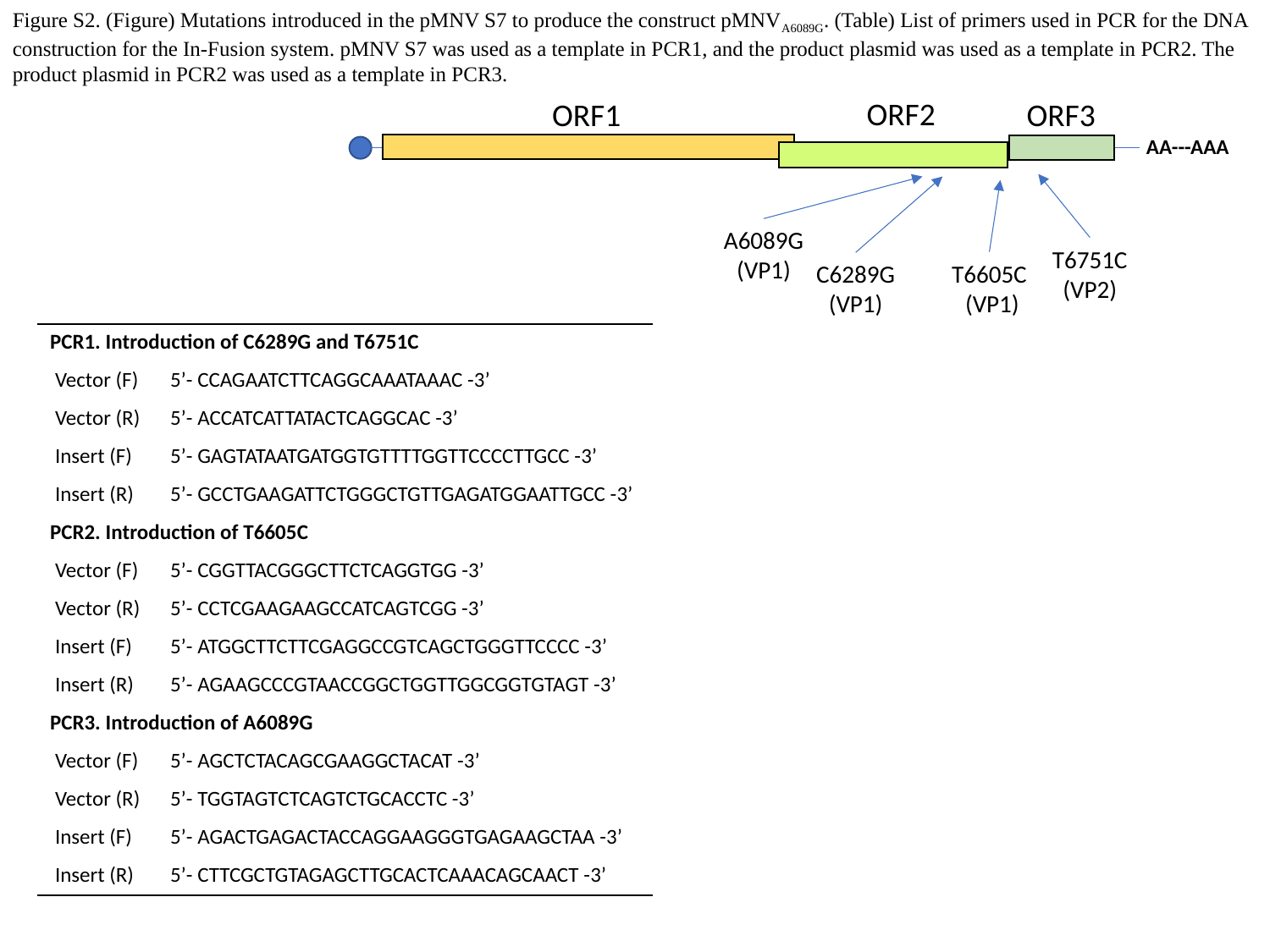

Figure S2. (Figure) Mutations introduced in the pMNV S7 to produce the construct pMNVA6089G. (Table) List of primers used in PCR for the DNA construction for the In-Fusion system. pMNV S7 was used as a template in PCR1, and the product plasmid was used as a template in PCR2. The product plasmid in PCR2 was used as a template in PCR3.
ORF2
ORF3
ORF1
AA---AAA
A6089G
(VP1)
T6751C
(VP2)
T6605C
 (VP1)
C6289G
(VP1)
| PCR1. Introduction of C6289G and T6751C | |
| --- | --- |
| Vector (F) | 5’- CCAGAATCTTCAGGCAAATAAAC -3’ |
| Vector (R) | 5’- ACCATCATTATACTCAGGCAC -3’ |
| Insert (F) | 5’- GAGTATAATGATGGTGTTTTGGTTCCCCTTGCC -3’ |
| Insert (R) | 5’- GCCTGAAGATTCTGGGCTGTTGAGATGGAATTGCC -3’ |
| PCR2. Introduction of T6605C | |
| Vector (F) | 5’- CGGTTACGGGCTTCTCAGGTGG -3’ |
| Vector (R) | 5’- CCTCGAAGAAGCCATCAGTCGG -3’ |
| Insert (F) | 5’- ATGGCTTCTTCGAGGCCGTCAGCTGGGTTCCCC -3’ |
| Insert (R) | 5’- AGAAGCCCGTAACCGGCTGGTTGGCGGTGTAGT -3’ |
| PCR3. Introduction of A6089G | |
| Vector (F) | 5’- AGCTCTACAGCGAAGGCTACAT -3’ |
| Vector (R) | 5’- TGGTAGTCTCAGTCTGCACCTC -3’ |
| Insert (F) | 5’- AGACTGAGACTACCAGGAAGGGTGAGAAGCTAA -3’ |
| Insert (R) | 5’- CTTCGCTGTAGAGCTTGCACTCAAACAGCAACT -3’ |

## Slide 3
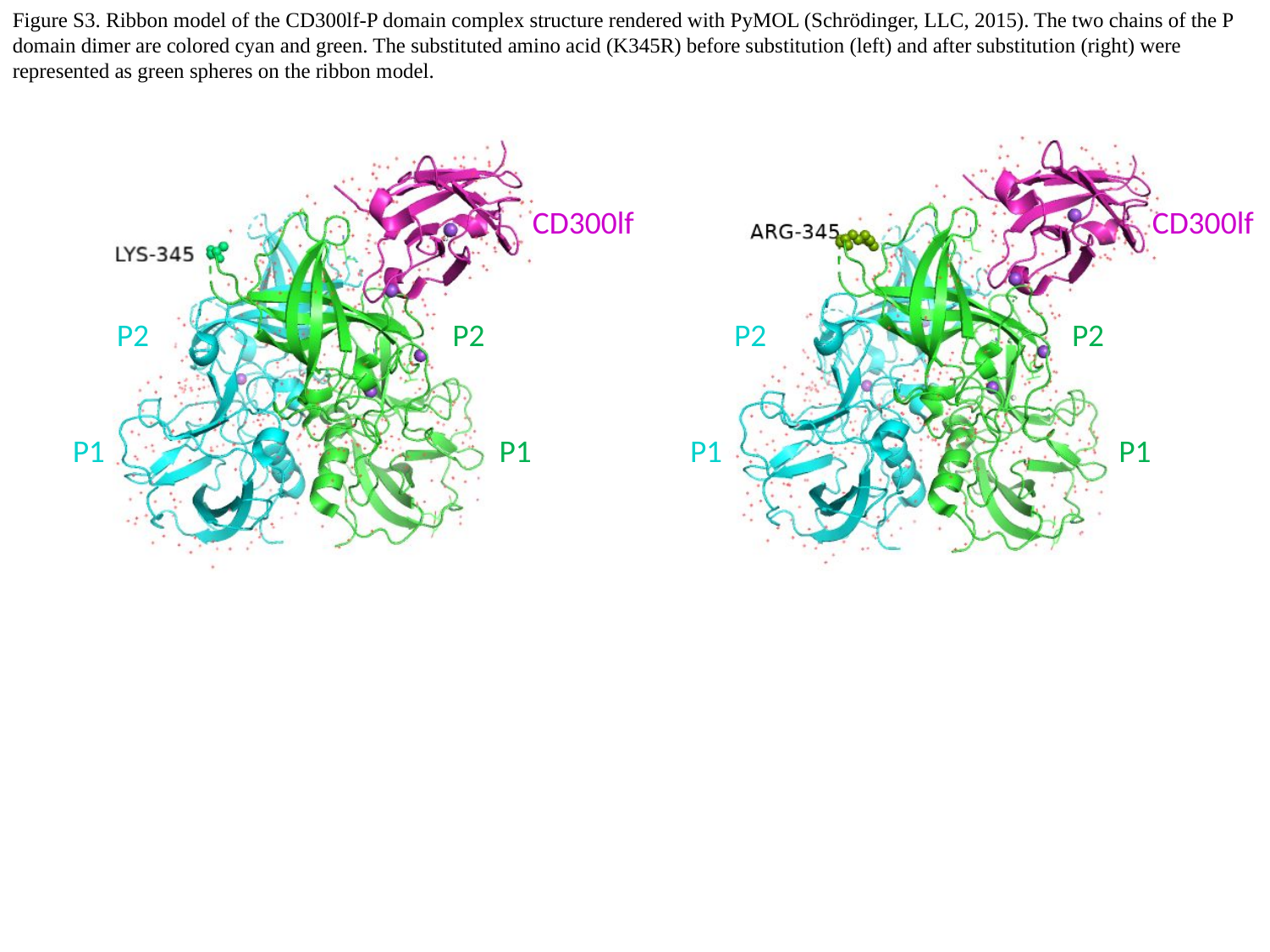

Figure S3. Ribbon model of the CD300lf-P domain complex structure rendered with PyMOL (Schrödinger, LLC, 2015). The two chains of the P domain dimer are colored cyan and green. The substituted amino acid (K345R) before substitution (left) and after substitution (right) were represented as green spheres on the ribbon model.
CD300lf
CD300lf
P2
P2
P2
P2
P1
P1
P1
P1

## Slide 4
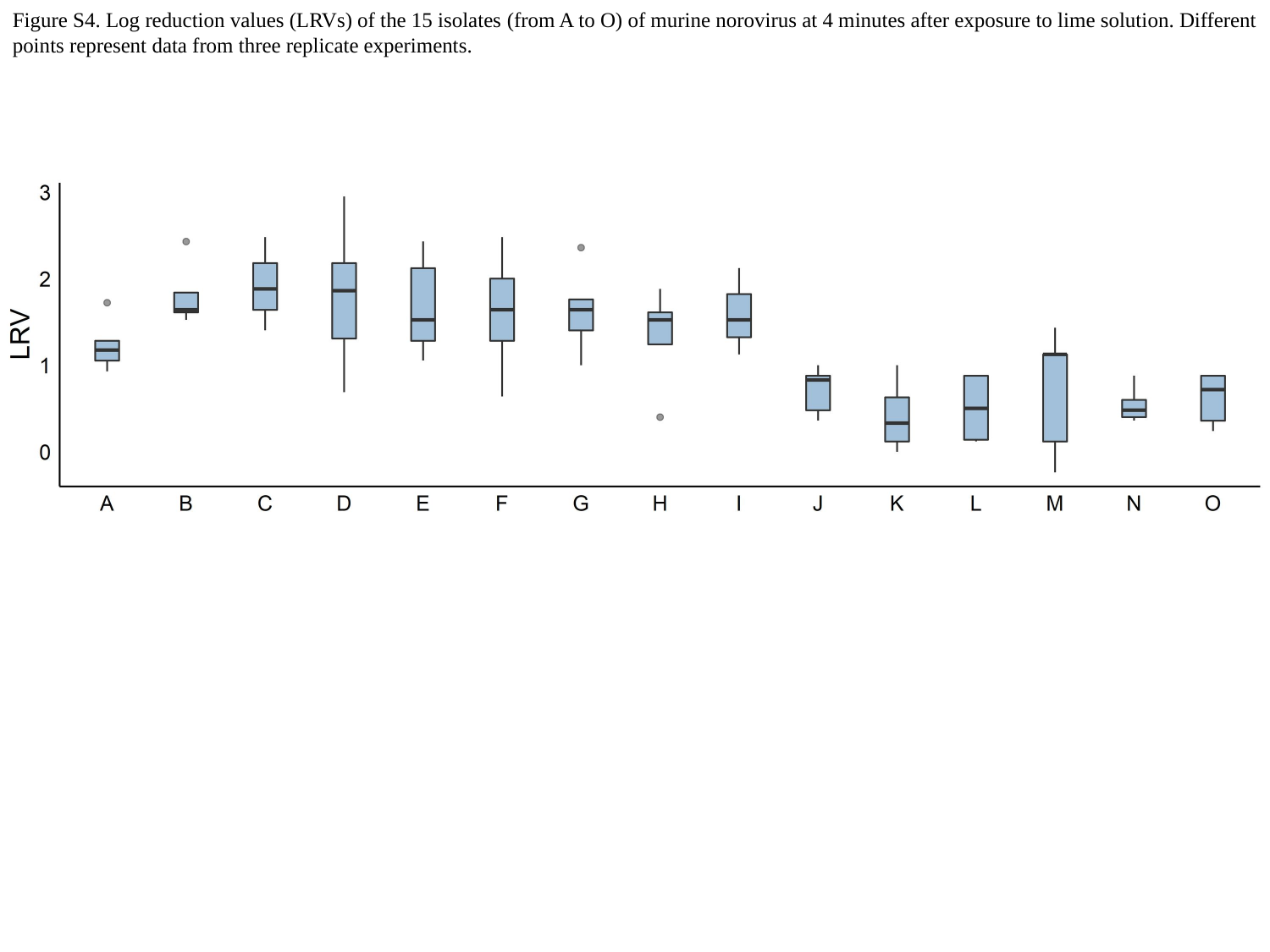

Figure S4. Log reduction values (LRVs) of the 15 isolates (from A to O) of murine norovirus at 4 minutes after exposure to lime solution. Different points represent data from three replicate experiments.

## Slide 5
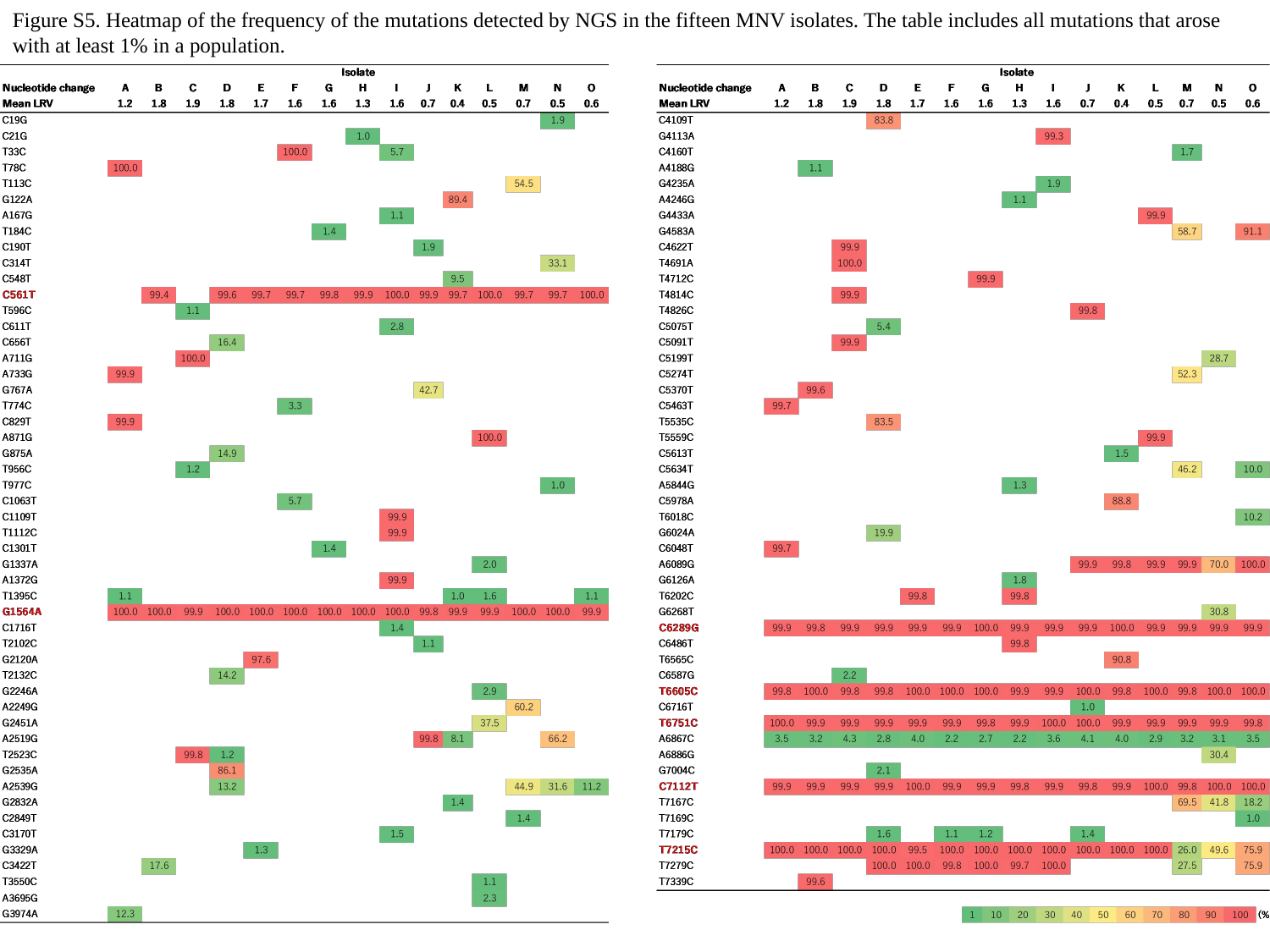

Figure S5. Heatmap of the frequency of the mutations detected by NGS in the fifteen MNV isolates. The table includes all mutations that arose with at least 1% in a population.

## Slide 6
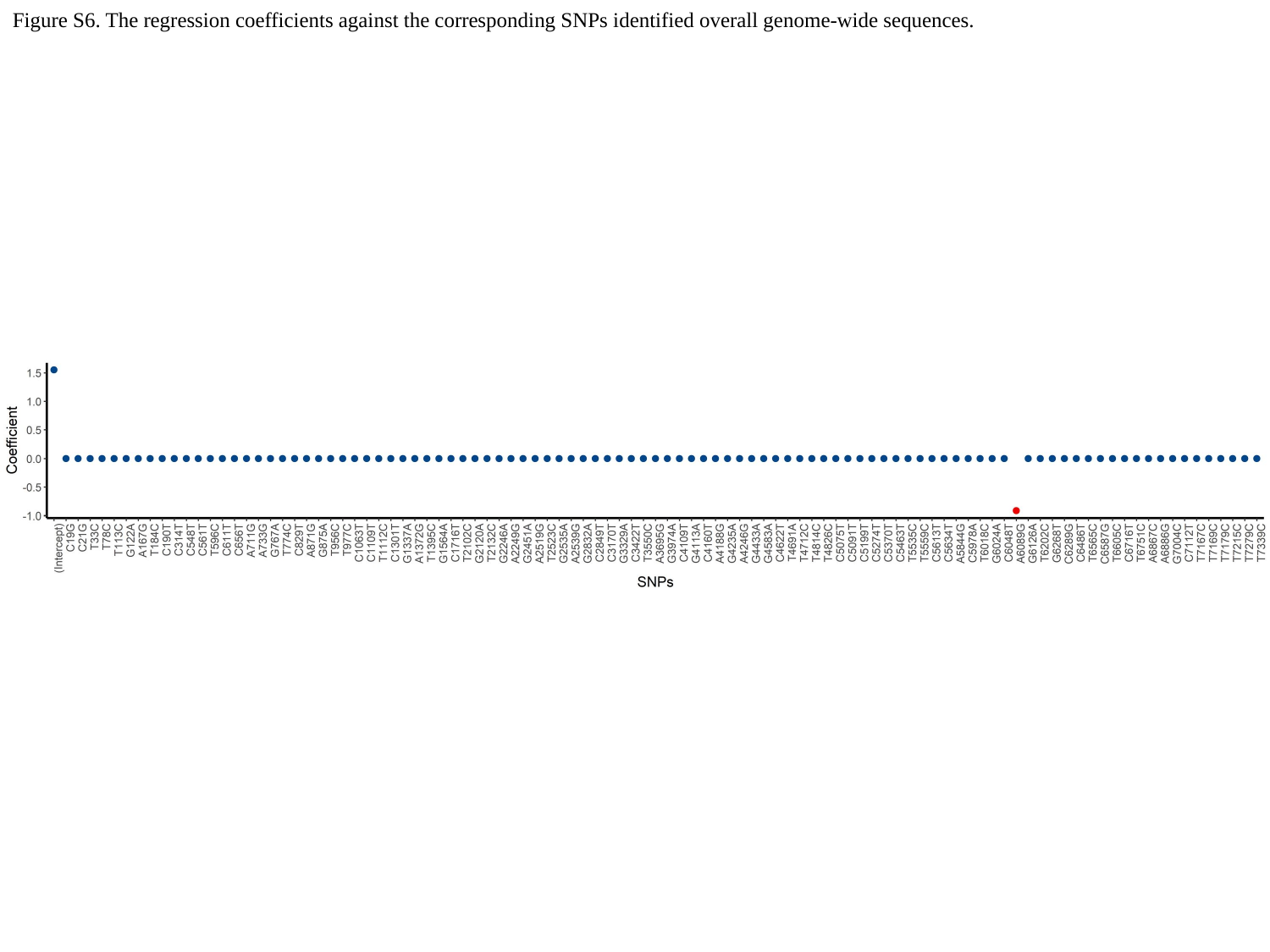

Figure S6. The regression coefficients against the corresponding SNPs identified overall genome-wide sequences.

## Slide 7
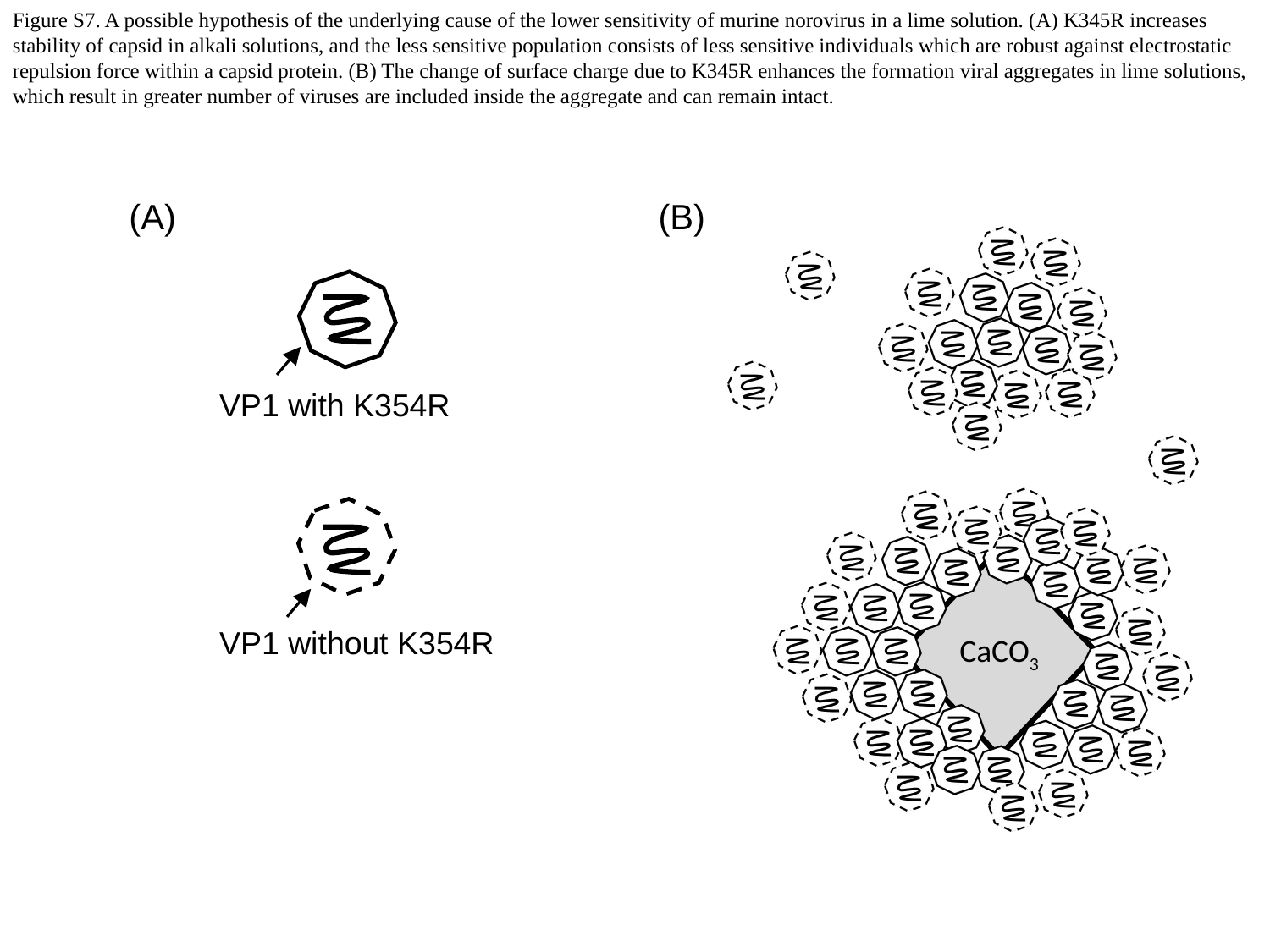

Figure S7. A possible hypothesis of the underlying cause of the lower sensitivity of murine norovirus in a lime solution. (A) K345R increases stability of capsid in alkali solutions, and the less sensitive population consists of less sensitive individuals which are robust against electrostatic repulsion force within a capsid protein. (B) The change of surface charge due to K345R enhances the formation viral aggregates in lime solutions, which result in greater number of viruses are included inside the aggregate and can remain intact.
(A)
(B)
VP1 with K354R
CaCO3
VP1 without K354R
